# Supplementation of heat-killed probiotics mixture improves intestinal morphology, antioxidant capacity, and meat quality in broilers

**DOI:** 10.1016/j.vas.2025.100462

**Published:** 2025-05-12

**Authors:** Bishnu Prasad Bhattarai, Fu-Yuan Cheng, Yu-Cheng Xu, Chi Yu, Ting-Yu Lee, Hsiao-Tung Chang, Hsiao-Ching Lin, Hsiu-Ming Weng, Hsin-Hsuan Huang, Jin-Seng Lin, Chao-Wei Huang

**Affiliations:** a Department of Tropical Agriculture and International Cooperation, National Pingtung University of Science and Technology, Pingtung, Taiwan; b Department of Animal Science, National Pingtung University of Science and Technology, Pingtung, Taiwan; c Culture Collection & Research Institute, SYNBIO TECH INC., Kaohsiung, Taiwan

**Keywords:** Heat-killed probiotics, Broilers, Antioxidant, Meat quality, Pelleting conditions

## Abstract

Probiotics are widely used to improve intestinal health and production performance in the poultry industry. However, few studies have explored the effects of heat-killed probiotics. This study investigated the effects of a heat-killed probiotic mixture, comprising *Bacillus subtilis* (BS) and *Lactobacillus plantarum* 28 (LP28), on the growth performance, intestinal morphology, antioxidant capacity, and meat quality of broilers. A total of 300 Arbor Acres chicks were randomly assigned to four treatment groups (three replicates per group; 25 chicks per replicate): CON (basal diet), AB (basal diet + 10 ppm enramycin), LDP (basal diet + heat-killed LP28 and BS, 1.0 × 10^8^ cells/kg each), and HDP (basal diet + heat-killed LP28 and BS, 1.0 × 10^9^ cells/kg each). Feed intake was recorded daily, and body weight was recorded at the end of each growth phase. Twelve birds per treatment were used for intestinal morphology and antioxidant capacity analyses, and eight birds were used for meat quality analysis on day 35. One-way ANOVA followed by Tukey test was performed to perform statistical comparison among groups at *p* < 0.05. No significant intergroup difference was observed in growth performance. However, villus height and crypt depth were higher in the HDP group than in the CON and LDP groups (*p* < 0.05). The HDP group exhibited a stronger antioxidant capacity, higher hepatic glutathione peroxidase levels in the liver, and lower thiobarbituric acid reactive substance levels in breast meat than did the CON group (*p* < 0.05). The HDP group also exhibited better meat quality with lower shear force and higher gumminess and chewiness than did the CON group (*p* < 0.05). In summary, heat-killed probiotics may improve the intestinal morphology, antioxidant capacity, and meat quality of broilers and can be utilized in the poultry industry.

## 1 Introduction

Probiotics are living microorganisms that improve intestinal health and gut-related immunity in the host (Fuller, 1992). These microorganisms can sustain intestinal integrity, enhance nutrient degradation and absorption, prevent pathogen colonization, modulate the immune system, and improve growth performance in broilers (Schoster et al., 2013; Aljumaah et al., 2020). Probiotics exert antioxidant effects through free radical scavenging, enzyme regulation, metal ion chelation, and microbial modulation, which in turn reduce oxidative stress and lipid peroxidation in muscle tissue (Bai et al., 2017; Feng & Wang, 2020). Therefore, probiotics may be used to improve meat quality by modifying the pH, color, and tenderness of meat (Bai et al., 2017; Xu et al., 2021). Live probiotics can substantially improve the growth performance, intestinal health, and meat quality of broilers (Mohammed et al., 2024; Peng et al., 2016). Despite the benefits of live probiotics, their effects on the host may vary depending on their formulation, feed preparation method, and ability to withstand the harsh conditions of the host’s intestine (Morgan et al., 2014).

Compared with mash feed, pelletized feed can markedly improve broiler performance—for example, by increasing body weight (BW) gain, feed intake, and feed conversion ratio (FCR) (Kamboh, 2016; Kuleile & Molapo, 2019). Therefore, many feed manufacturers tend to maximize temperature, pressure, and retention time to pelletize feed (McCracken, 2002). In current industrial practice, feed manufacturers tend to adopt conditioning temperatures of up to 90 °C to minimize foodborne pathogens, such as *Salmonella* sp. (Doyle & Erickson, 2006).

To ensure the viability of live probiotics, the production temperature and steam pressure should be maintained below 65 °C and 80 psi, respectively, during pelleting procedures (Monteiro et al., 2020). Compared with live probiotics, heat-killed probiotics have a prolonged shelf life and are not considerably affected by pelleting procedures, which highlights their potential for use in feed manufacturing (Piqué et al., 2019). Heat-killed probiotics tend to retain their fundamental characteristics and can release specific bacterial components, such as lipoteichoic acids and peptidoglycans, which contribute to intestinal function and immunomodulation (Incharoen et al., 2019; Khonyoung & Yamauchi, 2012). Therefore, heat-killed probiotics may serve as a reliable feed additive in poultry production because they can withstand adverse temperature and pressure conditions during feed preparation.

Nonpathogenic gram-positive bacteria are the most commonly used probiotics in poultry production. These bacteria include *Bacillus* spp., *Bifidobacterium* spp., and lactic acid bacteria. *Bacillus* and *Lactobacillus* are widely used in the animal industry. *Bacillus subtilis* (BS) is a gram-positive, rod-shaped bacterium capable of sporulation, which enables a long shelf life during pelleting and facilitates storage across a wide temperature range (Chaiyawan et al., 2010). *Lactobacillus plantarum* is a gram-positive, catalase-negative, lactic acid bacterium. It is primarily used as a growth promoter and immunostimulant in animal production (Yang et al., 2016). Lactic acid bacteria are usually sensitive to temperatures exceeding 50 °C, and their thermal tolerance range varies depending on species and strain Aguinaga Bósquez et al. (2022). Despite this sensitivity, heat-killed L. *plantarum* exhibit resistance to adverse pelleting conditions; moreover, its inclusion in feed significantly (*p* < 0.05) improves the BW gain, feed performance, cytokine gene expression, and intestinal health of broilers (Incharoen et al., 2019).

In poultry production, the efficacy of probiotics as feed additives depends on their stability under harsh manufacturing conditions, storage duration, and effects on broiler performance. Few studies have explored the effects of heat-killed BS and L. *plantarum* on broilers. Therefore, this study investigated the effects of a heat-killed probiotic mixture, composed of equal proportions of BS and L. *plantarum* 28 (LP28), on the growth performance, intestinal morphology, antioxidant capacity, and meat quality of Arbor Acres broilers.

## 2 Materials and methods

This study was conducted in a research farm at National Pingtung University of Science and Technology, Taiwan. All animal experimental protocols were reviewed and approved by the Institutional Animal Care and Use Committee of National Pingtung University of Science and Technology (approval no NPUST-111–083).

### 2.1 Experimental Design and treatments

The experiment was conducted in a broiler barn equipped with tunnel ventilation and water-cooling pads over a period of 35 days. A total of 300 Arbor Acres chicks aged 1 day were randomly allocated to 15 floor pens (2 *m* × 1.7 *m* × 2 m). These chicks were divided into four dietary treatment groups (three replicates per group; 25 chicks per replicate): CON (basal diet), AB (basal diet supplemented with 10 ppm enramycin), LDP (basal diet supplemented with heat-killed LP28 and BS, each at a concentration of 1.0 × 10^8^ cells/kg), and HDP (basal diet supplemented with heat-killed LP28 and BS, each at a concentration of 1.0 × 10^9^ cells/kg). All chicks were provided with water and feed ad libitum. In accordance with the guidelines for managing Arbor Acres broilers, the chicks were maintained in an intermittent lighting cycle of 30 lx—23 h of light and 1 h of darkness—from day 0 to day 7 and 10 lx—20 h of light and 4 h of darkness—from day 8 to day 35. The experimental period was divided into three feeding phases: a starter phase (from day 1 to day 10), a grower phase (from day 11 to day 24), and a finisher phase (from day 25 to day 35). The average temperature and relative humidity were maintained at, respectively, 27.28 °*C* ± 1.81 °C and 70.33 % ± 11.74 % in the starter phase, 26.71 °*C* ± 1.35 °C and 82.93 % ± 6.28 % in the grower phase, and 27.44 °*C* ± 0.57 °C and 86.77 % ± 4.16 % in the finisher phase. Finally, growth performance, intestinal morphology (jejunum samples), antioxidant capacity (serum and liver samples), and meat quality (breast meat samples) were evaluated to determine the effects of probiotics.

### 2.2 Probiotic Preparation and experimental diets

Probiotic mixtures containing LP28 and BS were purchased from SYNBIO TECH INC. (Kaohsiung, Taiwan). Heat-killed probiotics were prepared in accordance with a method described by Wagner et al. (2000), with some modifications. In brief, a probiotic powder was spread on a sealed aluminum foil bag and autoclaved at 121 °C for 15 min. Aliquots were then cultured on modified de Man–Rogosa–Sharpe and plate count agar plates. The absence of cultivable bacteria in the suspensions confirmed that the probiotics were heat-killed. The experimental basal diet was based on corn–soybean meal and was formulated in accordance with the nutritional guidelines for Arbor Acres broilers (Table 1). Feed additives—enramycin and heat-killed probiotics—were added to the basal diet at specific concentrations. Mash feed was provided to all treatment groups during the starter phase, whereas pellet feed was provided during the grower and finisher phases.

**Table 1:** Chemical composition of the experimental basal diets (% as fed basis during the three experimental phases).

Items^1^	Diet		
	Starter (0–10 d)	Grower (11–24 d)	Finisher (25 −35 d)
Ingredient (%)			
Corn	48.45	52.40	57.47
Soybean meal (45 %)	42.50	38.40	33.10
Soybean oil	4.60	5.25	5.80
CaHPO_4_	2.84	2.55	2.30
Limestone	0.40	0.35	0.33
Met (98 %)	0.35	0.30	0.30
Lys	0.10	0.10	0.10
Thr	0.06	0.05	0.00
Vitamin premix a	0.20	0.15	0.15
Minerals premix b	0.20	0.15	0.15
NaCl	0.30	0.30	0.30
Total	100	100	100
calculated composition			
Crude protein (%)	23.00	21.50	19.50
ME kcal/kg	3000.00	3100.00	3200.00
Ca%	0.96	0.87	0.79
Available P%	0.48	0.44	0.40
Digestible Met + Cys %	0.95	0.87	0.80
Digestible Lys %	1.28	1.15	1.03
Digestible Trp %	0.20	0.18	0.16
Digestible Thr %	0.86	0.77	0.69

1 d: days, CaHPO_4_: Dicalcium Phosphate, NaCl: Sodium Chloride, ME: Metabolizable Energy, Ca: Calcium, Met: Methionine, Cys: cysteine, Lys: Lysine, Trp: Tryptophan and Thr: Threonine.

a Vitamin premix provided per kilogram of diet: vitamin A, 10,000,000 IU; vitamin D3, 1000,000 IU; vitamin E, 20 g; vitamin K3, 1.5 g; Vitamin B, 2 g; vitamin B2, 4 g; vitamin B6, 3 g; vitamin B12, 15 mg; biotin, 60 mg; pantothenic acid, 15 g; folic acid, 1 g; and niacin (Niacin): 35 g.

b Mineral premix provided per kilogram of diet: cobalt, 100 mg; copper, 5 g; iodine, 400 mg; iron, 40 g; manganese, 60 g; selenium, 300 mg; zinc, 40∼44 g; lead, 50 ppm; mercury, 0.5 ppm; cadmium, 10 ppm; and arsenic, 12 ppm.

### 2.3 Growth Performance

Feed offered and feed refusal were measured daily by using an electronic scale to calculate the average daily feed intake (ADFI; g/chicken) in each phase. All broilers were individually weighed using an electronic scale on days 1, 14, 21, and 35 without premeasurement feed restriction to determine their average daily weight gain (ADWG; g/chicken). In addition, the FCR (feed [g]/weight gain [g]) in each treatment group was calculated during each phase.

### 2.4 Intestinal Morphology

On day 35, jejunum samples were collected from 12 broilers from each treatment group to examine intestinal morphology following the method of Gava et al. (2015), with some modifications. In brief, 2 to 3 cm of the jejunum was transversely cut, gently rinsed with phosphate-buffered saline, and stored in a 10 % buffered formalin solution. After the samples were embedded in paraffin, they were sliced to a thickness of 3 to 4 μm and subjected to hematoxylin–eosin staining. Villus height (VH) was measured as the vertical distance from the villus tip to the villus–crypt junction. Crypt depth (CD) was measured as the vertical distance from the villus–crypt junction to the lower limit of the crypt. Images were captured using a Nikon microscope (Nikon Eclipse 80i; Nikon, Tokyo, Japan) connected to an integrated digital imaging analysis system. A total of 10 villi per sample were examined. An average of 10 villi obtained from 12 samples per treatment group were used for statistical analysis.

### 2.5 Antioxidant Capacity in plasma and liver

On day 35, blood samples were obtained by puncturing the brachial vein and collecting blood in heparinized tubes for immunological analysis (*n* = 12). Each sample was centrifuged at 3000 ×*g* for 15 min for plasma isolation. Then, the plasma was stored at −20 °C until further analysis (Xu et al., 2021). After the broilers were euthanized, liver tissue was collected and immediately placed in liquid nitrogen. Subsequently, liver tissue was homogenized in 9 mL of 0.9 % sodium chloride buffer at 8000 rpm for 10 s on ice and centrifuged at 3500 rpm for 15 min at 4 °C. The supernatant of the liver homogenization solution was stored at −20 °C for further analysis (Bai et al., 2017). The plasma and liver supernatant solutions were used to examine the activity of superoxide dismutase (SOD), catalase, and glutathione peroxidase (GPx) by using commercially available kits.

### 2.6 Meat Quality

All broilers were transported in crates (0.73 *m* × 0.54 *m* × 0.26 m) over a distance of 16 km from the farm to the slaughterhouse (transportation duration: 25 min). Eight broilers were selected from each treatment group. The average transportation temperature and relative humidity on the day of the experiment were 29 °C and 79 %, respectively. After the broilers were slaughtered in the slaughterhouse’s production line, breast meat was collected from each broiler after 24 h to examine its physiochemical characteristics. Minced breast meat and distilled water were mixed at a ratio of 1:10 and homogenized for 1 min. Subsequently, a pH meter (model SP-2100; Suntex, Taipei, Taiwan) was used to measure pH as described by Troutt et al. (1992), with minor modifications. Each measurement was repeated thrice, and the average pH value was recorded. Meat color—represented as breast lightness (L*), redness (a*), and yellowness (b*)—was evaluated using a Chroma meter (CR-400; Konica Minolta, Tokyo, Japan) at three different points on the top side of each breast meat sample, and the average value of meat color was recorded (Al-Owaimer et al., 2014). Carcasses were stored at 4 °C for 24 h in plastic bags, and the difference between the initial and final weight was determined to calculate drip loss (DL), expressed as a percentage (Al-Owaimer et al., 2014). Breast meat samples were cooked in a water bath (80 °C) until their internal temperature reached 75 °C (approximately 40 min), and the difference in weight before and after cooking was determined to calculate cooking loss (CL), expressed as a percentage (Liao et al., 2022). Subsequently, cooked breast meat was trimmed into three pieces (length: 4 cm; width: 4 cm; height: 2 cm), which were used to measure shear force following the method of Hernández-Coronado et al. (2019), with some modifications. A texture profile analyzer (TA.XTplusC; Stable Micro Systems, Surrey, UK) connected to a computer and equipped with an A/MORS blade was operated at a trigger force of 50 g, a compression depth of 10 mm, and a compression speed of 5 mm/s to measure the shear force at three different points, spaced 1 cm apart, on each sample; the average value was recorded. The texture profiles of cooked samples were analyzed after adjustment following the method of Zhuang and Savage (2012). Each sample was trimmed to pieces measuring 4 cm in length, 4 cm in width, and 2 cm in height. The same texture profile analyzer was used to measure the hardness, springiness, cohesiveness, gumminess, and chewiness of each sample (Liao et al., 2022). Measurements were taken twice by using a P/1S ball-type measuring probe at a trigger force of 30 g, a depression depth of 10 mm, and a probe descending speed of 5 mm/s. Water holding capacity (WHC) was determined using the method outlined by Musa et al. (2006), with minor modifications. In brief, 1 g of breast meat was placed on a filter paper (No. 1; Adventec), covered with acetate paper, and pressed at 3000 psi for 1 min using a pressure gauge (Model C; Carver, USA). After drying, the surface areas of the inner and outer rings were measured using a planimeter (KP-90 N; KOIZUMI, Japan). Subsequently, the WHC was calculated as the ratio of the difference between the surface areas of the outer and inner rings to the surface area of the inner ring, and it was expressed as a percentage. Next, 5 g of each breast sample was mixed with 10 mL of 20 % trichloroacetic acid and 10 mL of distilled water and homogenized at 10,000 rpm for 30 s to measure thiobarbituric acid (TBA) reactive substances (TBARS). After the homogenate was centrifuged at 1401 ×*g* for 10 min, it was filtered using Whatman No 1 filter paper. Subsequently, 2 mL of the supernatant was added to 2 mL of 0.02 mol/L TBA in a test tube. After the tube was heated at 100 °C for 30 min, it was allowed to cool down under running water for 10 min. Meanwhile, a blank solution was prepared using 1 mL of 20 % trichloroacetic acid, 1 mL of distilled water, and 2 mL of 0.02 mol/L TBA. Next, the absorbance of the resulting solution was measured at 530 nm by using a microplate reader (SPECTROstar Nano; BMG LABTECH, Germany). Finally, a standard curve was obtained using ethoxypropane (1,1,3,3-tetraethoxypropane, T9889; Sigma-Aldrich, USA), and TBARS were expressed as milligrams of malondialdehyde (MDA) per kilogram of muscle (Faustman et al., 1992).

### 2.7 Statistical Analysis

Data were analyzed using one-way analysis of variance. The analyses were performed using SPSS (version 22.0; IBM, Armonk, NY, USA). Statistical significance was set at *p* < 0.05. Tukey’s honestly significant difference test was performed in cases of significant intergroup differences. Nonnumerical parameters were compared using the Kruskal–Wallis test. Data are presented as mean ± standard error of the mean (SEM).

## 3 Results

### 3.1 Growth Performance

Growth performance was evaluated during the three phases of feeding. During the starter phase, the AB group had a significantly higher BW and ADWG than did the other groups, whereas the LDP group had a significantly lower BW and ADWG than did the other groups (*p* < 0.05; Table 2). In addition, the LDP group had a significantly lower ADFI than did the other groups (*p* < 0.05). Moreover, the AB group had a significantly lower FCR than did the other groups, whereas the LDP group had a significantly higher FCR than did the other groups (*p* < 0.05).

**Table 2:** Effects of different concentrations of heat-killed probiotics on growth performance of broiler chickens^1^.

Items^2^	Treatments^3^			
	CON	AB	LDP	HDP
BW, g/bird				
1 d	41.46 ± 0.35	40.91 ± 0.31	41.31 ± 0.33	41.78 ± 0.33
14 d	413.65 ± 4.43 ^b^	444.92 ± 4.86 ^a^	364.32 ± 4.18 ^c^	418.22 ± 3.47 ^b^
21 d	884.76 ± 15.01 ^BCE^	947.83 ± 10.76 ^a^	849.68 ± 8.31 ^c^	911.15 ± 8.92 ^b^
35 d	2093.25 ± 24.71	2110.09 ± 25.44	2047.76 ± 17.07	2126.08 ± 24.06
ADWG, g/ bird				
1 - 14 d	26.59 ± 0.32 ^b^	28.86 ± 0.34 ^a^	23.07 ± 0.29 ^c^	26.89 ± 0.25 ^b^
15 - 21 d	67.30 ± 1.97 ^b^	71.84 ± 0.99 ^a^	69.34 ± 0.73 ^ab^	70.42 ± 0.87 ^ab^
22 - 35 d	86.32 ± 1.67	83.02 ± 1.21	85.58 ± 0.90	86.78 ± 1.26
1 - 35 d	58.62 ± 0.71	59.12 ± 0.72	57.33 ± 0.49	59.55 ± 0.69
ADFI, g/bird				
1 - 14 d	35.12 ± 0.16 ^a^	35.83 ± 0.13 ^a^	33.15 ± 0.41 ^b^	35.52 ± 0.14 ^a^
15 - 21 d	90.58 ± 0.45 ^ab^	92.14 ± 1.24 ^a^	87.60 ± 1.24 ^b^	92.63 ± 0.28 ^a^
22 - 35 d	135.09 ± 1.87	130.46 ± 2.97	129.73 ± 1.45	134.95 ± 1.42
1 - 35 d	86.20 ± 0.88	84.94 ± 1.47	82.67 ± 0.92	86.71 ± 0.46
FCR, g/g				
1 - 14 d	1.321 ± 0.021 ^b^	1.242 ± 0.010 ^c^	1.437 ± 0.003 ^a^	1.320 ± 0.004 ^b^
15 - 21 d	1.347 ± 0.023	1.284 ± 0.027	1.263 ± 0.018	1.315 ± 0.013
22 - 35 d	1.565 ± 0.022	1.573 ± 0.031	1.516 ± 0.025	1.556 ± 0.009
1 - 35 d	1.470 ± 0.015	1.438 ± 0.025	1.442 ± 0.020	1.456 ± 0.020

1 n=3. ^abc^Mean values ± standard error of the mean (SEM) in the same row with a different superscript differ significantly (*p<0.05*).

2 BW, body weight; ADWG, Average daily weight gain; ADFI, Average daily feed intake; FCR, Feed conversion ratio.

3 CON, birds fed the basal diet; AB, birds fed the basal diet with 10 ppm Enramycin; LDP, basal diet supplemented with heat-killed probiotics, including L. *plantarum* LP28 and *B. subtilis*, each at a concentration of 1.0 × 10^8^ cells/kg, and HDP, basal diet supplemented with heat-killed probiotics, including L. *plantarum* LP28 and *B. subtilis*, each at a concentration of 1.0 × 10^9^ cells/kg.

During the grower phase, the AB group had a significantly higher BW than did the other groups (*p* < 0.05). On day 21, the LDP group had a significantly lower BW than did the AB and HDP groups (*p* < 0.05). No difference in ADWG was observed between the LDP and HDP groups or between the CON and AB groups. However, the AB group had a significantly higher ADWG than did the CON group (*p* < 0.05). In addition, the LDP group had a significantly lower ADFI than did the AB and HDP groups (*p* < 0.05). Nonetheless, no significant intergroup difference was observed in FCR (Table 2).

During the finisher phase, no significant intergroup difference was noted in BW, ADWG, ADFI, or FCR were observed.

Overall, no significant intergroup difference was observed in BW, ADWG, ADFI, or FCR during the study period (from day 1 to day 35; Table 2).

### 3.2 Intestinal Morphology

VH was significantly higher in the HDP group (1125.49 µm) than in the CON (974.37 µm), AB (1100.49 µm), and LDP (1017.98 µm) groups (*p* < 0.05; Table 3). CD was significantly higher in the LDP and HDP groups (226.60 and 254.32 µm, respectively) than in the CON and AB groups (187.66 and 199.27 µm, respectively; *p* < 0.05). However, the VH-to-CD ratio was significantly lower in the LDP and HDP groups (4.72 and 4.58, respectively) than in the CON and AB groups (5.40 and 5.87, respectively; *p* < 0.05).

**Table 3:** Effects of different concentrations of heat-killed probiotics on the intestinal morphological alterations of broiler chickens at day 35^1^.

Items^2^	Treatments^3^			
	CON	AB	LDP	HDP
VH, µm	974.37 ± 19.98 ^b^	1100.49 ± 14.89 ^a^	1017.98 ± 16.39 ^b^	1125.49 ± 26.04 ^a^
CD, µm	187.66 ± 4.48 ^c^	199.27 ± 5.04 ^c^	226.60 ± 5.59 ^b^	254.32 ± 6.95 ^a^
VH: CD	5.44 ± 0.16 ^a^	5.87 ± 0.19 ^a^	4.72 ± 0.12 ^b^	4.58 ± 0.13 ^b^

1 n=12. ^abc^Mean values ± standard error of the mean (SEM) in the same row with a different superscript differ significantly (*p<0.05*).

2 VH, villus height; CD, Crypt depth; VH-to-CD ratio, Villus height to crypt depth ratio.

3 CON, birds fed the basal diet; AB, birds fed the basal diet with 10 ppm Enramycin; LDP, basal diet supplemented with heat-killed probiotics, including L. *plantarum* LP28 and *B. subtilis*, each at a concentration of 1.0 × 10^8^ cells/kg, and HDP, basal diet supplemented with heat-killed probiotics, including L. *plantarum* LP28 and *B. subtilis*, each at a concentration of 1.0 × 10^9^ cells/kg.

Fig. 1

**Fig. 1:**
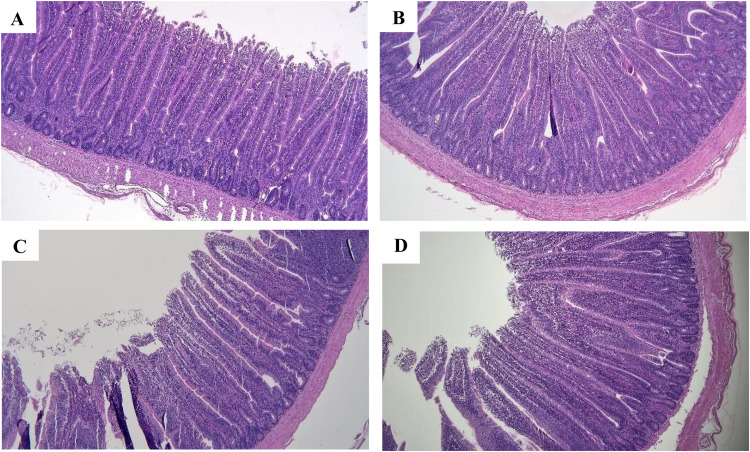
Histological representations of jejunum tissue from experimental broiler chickens at day 35 under original magnification (× 100). A: Control group; basal diet, B; AB group, basal diet with 10 ppm enramycin; C: LDP, basal diet supplemented with heat-killed probiotics, including L. *plantarum* LP28 and *B. subtilis*, each at a concentration of 1.0 × 10^8^ cells/kg, and HDP, basal diet supplemented with heat-killed probiotics, including L. *plantarum* LP28 and *B. subtilis*, each at a concentration of 1.0 × 10^9^ cells/kg.

### 3.3 Antioxidant Capacity

**Fig. 2:**
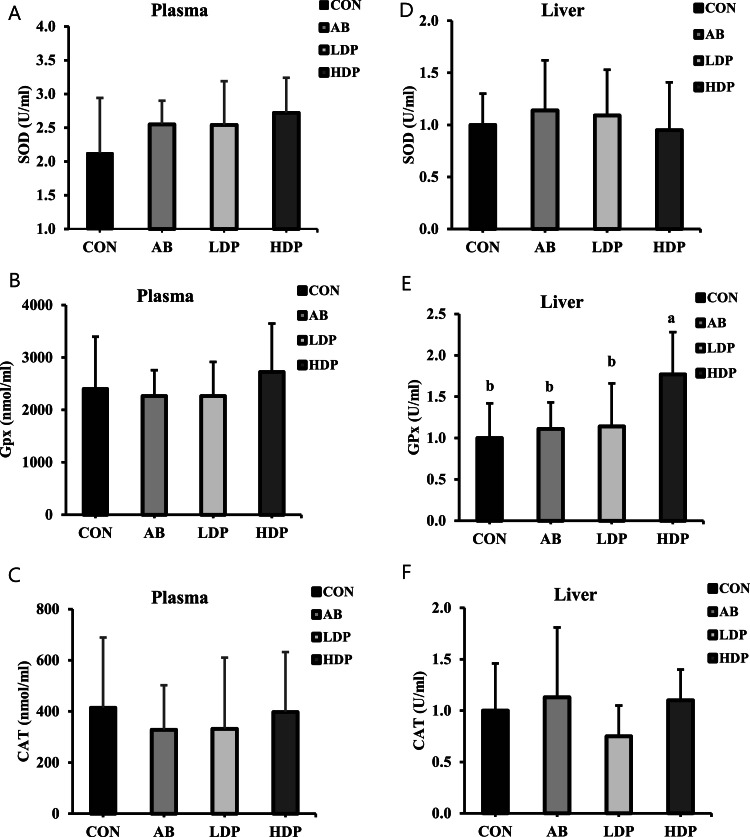
The effects of different concentrations of heat-killed probiotics on antioxidant enzymes level in the serum (A-C) and liver (D-F) of broilers chicken at day 35. ^ab^Mean values and standard error of the mean (SEM) with a different superscript differ significantly at *p* < 0.05. SOD, Superoxide dismutase; GPx, Glutathione peroxidase; and CAT, Catalase. CON, birds fed the basal diet; AB, birds fed the basal diet with 10 ppm Enramycin; LDP, basal diet supplemented with heat-killed probiotics, including L. *plantarum* LP28 and *B. subtilis*, each at a concentration of 1.0 × 10^8^ cells/kg, and HDP, basal diet supplemented with heat-killed probiotics, including L. *plantarum* LP28 and *B. subtilis*, each at a concentration of 1.0 × 10^9^ cells/kg.

Analysis of the liver samples revealed that the HDP group had significantly higher GPx levels than did the other groups (*p* < 0.05; Fig. 2E). However, analysis of both the serum and liver samples revealed no significant intergroup differences in the level of SOD or catalase; moreover, no significant difference was observed in the level of GPx in serum (Figs. 2A–2D and 2F). Additionally, TBARS levels were significantly lower in the AB, LDP, and HDP groups (0.74, 0.64, and 0.64 mg MDA, respectively) than in the CON group (1.05 mg MDA, *p* < 0.05; Table 4).

**Table 4:** Effects of different concentrations of heat-killed probiotics on physical characteristics of breast meat in broilers^1^.

Items^2^	Treatments^3^			
	CON	AB	LDP	HDP
pH	6.17 ± 0.04	6.09 ± 0.04	6.10 ± 0.04	6.07 ± 0.03
L*	62.44 ± 0.50	62.71 ± 0.45	62.42 ± 0.26	62.27 ± 0.45
a*	2.47 ± 0.16 ^a^	1.58 ± 0.06 ^b^	1.82 ± 0.18 ^b^	1.97 ± 0.18 ^ab^
b*	7.64 ± 0.32 ^ab^	7.56 ± 0.25 ^ab^	8.45 ± 0.14 ^a^	7.21 ± 0.25 ^b^
CL, %	29.08 ± 1.62	27.84 ± 1.44	28.00 ± 1.75	29.26 ± 0.79
DL, %	0.62 ± 0.06	0.52 ± 0.06	0.61 ± 0.07	0.58 ± 0.07
WHC, %	53.67 ± 0.97	52.00 ± 1.02	51.57 ± 1.55	51.83 ± 1.38
TBARS, mg MDA/kg	1.05 ± 0.12 ^a^	0.74 ± 0.10 ^b^	0.64 ± 0.07 ^b^	0.64 ± 0.05 ^b^

1 n=8. ^ab^ Mean values ± standard error of the mean (SEM) in the same row with a different superscript differ significantly (*p<0.05*).

2 L*: Lightness, a*: redness, b*: yellowness, CL: cooking loss, DL: Dripping loss, WHC: water holding capacity; TBARS, Thiobarbituric acid reactive substances.

3 CON, birds fed the basal diet; AB, birds fed the basal diet with 10 ppm Enramycin; LDP, basal diet supplemented with heat-killed probiotics, including L. *plantarum* LP28 and *B. subtilis*, each at a concentration of 1.0 × 10^8^ cells/kg, and HDP, basal diet supplemented with heat-killed probiotics, including L. *plantarum* LP28 and *B. subtilis*, each at a concentration of 1.0 × 10^9^ cells/kg.

### 3.4 Meat Quality

The effects of probiotic supplementation on the physical characteristics of breast meat were examined on day 35 (Table 4). No significant intergroup difference was noted in pH, L*, CL, DL, or WHC. However, the a* value significantly higher in the CON group (2.47) than in the AB (1.58) and LDP (1.82) groups (*p* < 0.05). In addition, the b* value was significantly lower in the HDP group (7.21) than in the LDP group (8.45; *p* < 0.05).

Texture profile analysis revealed that shear force values were significantly lower in the LDP and HDP groups (837.12 and 858.49 g, respectively) than in the CON group (*p* < 0.05; Table 5). Gumminess was significantly higher in the LDP and HDP groups (1348.76 and 1225.30 g, respectively) than in the CON group (1078.60 g). Chewiness was significantly higher in the LDP and HDP groups (1140.44 and 1089.30 g, respectively) than in the CON and AB groups (828.54 and 932.73 g, respectively; *p* < 0.05; Table 5). However, probiotic supplementation exerted no significant effect on the hardness, springiness, or cohesiveness of breast meat.

**Table 5:** Effects of different concentrations of heat-killed probiotics on texture profile analysis of the broilers’ breast meat^1^.

Items	Treatments^2^			
	CON	AB	LDP	HDP
Shear value, N	982.25 ± 24.48 ^a^	877.79 ± 22.44 ^b^	837.12 ± 25.11 ^b^	858.49 ± 20.87 ^b^
Hardness, N	1577.55 ± 48.95	1706.43 ± 50.29	1663.05 ± 69.05	1712.83 ± 51.15
Springiness, cm	0.76 ± 0.01	0.76 ± 0.01	0.79 ± 0.01	0.77 ± 0.01
Cohesiveness	0.70 ± 0.02	0.69 ± 0.02	0.69 ± 0.02	0.68 ± 0.02
Gumminess, N	1078.60 ± 12.25 ^c^	1173.13 ± 20.43 ^BCE^	1348.76 ± 34.58 ^a^	1225.30 ± 41.36 ^b^
Chewiness, N cm	828.54 ± 16.46 ^b^	944.09 ± 32.86 ^b^	1140.44 ± 30.95 ^a^	1089.30 ± 44.52 ^a^

1 n=8. ^ab^ Mean values ± standard error of the mean (SEM) in the same row with a different superscript differ significantly (*p<0.05*).

2 CON, birds fed the basal diet; AB, birds fed the basal diet with 10 ppm Enramycin; LDP, basal diet supplemented with heat-killed probiotics, including L. *plantarum* LP28 and *B. subtilis*, each at a concentration of 1.0 × 10^8^ cells/kg, and HDP, basal diet supplemented with heat-killed probiotics, including L. *plantarum* LP28 and *B. subtilis*, each at a concentration of 1.0 × 10^9^ cells/kg.

## 4 Discussion

In this study, heat-killed probiotics exerted no significant effect on broilers’ growth parameters—BW, ADWG, ADFI, or FCR—from day 1 to day 14. These findings are consistent with those of Reis et al. (2017), who reported that administering live BS at a concentration of 1.6 × 10^12^ CFU/kg did not have any significant effect on broilers’ growth parameters during the first week of treatment. Bai et al. (2017) indicated that administering live BS at a concentration ranging from 2 × 10^10^ to 4 × 10^10^ CFU/kg exerted no significant effect on BW, ADWG, ADFI, or FCR between days 1 and 21. During the early stages of growth, birds develop their intestinal integrity, and their gastrointestinal tract contains a small number of microorganisms (Reis et al., 2017). Therefore, administering probiotics may not improve their intestinal health or growth performance (Lutful Kabir, 2009). Therefore, probiotics have limited effects during the starter phase. Khonyoung and Yamauchi (2012) reported that administering 1 mg/kg heat-killed L. *plantarum*l-137 did not significantly improve growth performance. However, Incharoen et al. (2019) demonstrated that administering 10 mg/kg heat-killed L. *plantarum*l-137 significantly improved growth performance (*p* < 0.05). Together, these findings indicate that probiotics do not significantly alter growth performance; however, at appropriate concentrations, they help maintain growth performance during the early stage of supplementation. In the present study, no significant intergroup differences were observed in BW, ADWG, ADFI, or FCR. Reis et al. (2017) reported that administering live BS (DSM 17,299) at a concentration of 1.6 × 10^9^ CFU/kg did not alter the BW gain, feed intake, or FCR of Ross broilers. Similarly, Sampath et al. (2021) demonstrated that adding 0.10 % live L. *plantarum* to broilers’ diet resulted in no significant changes in their feed intake or FCR.

We examined intestinal morphology as an indicator of broilers’ intestinal health. The HDP group had a significantly higher VH than did the CON and LDP groups (*p* < 0.05). These findings are consistent with those of Song et al. (2014), who reported that administering high concentrations of live multistrain probiotics (i.e., *Bacillus licheniformis* at 1 × 10^10^ CFU/kg, BS at 1 × 10^10^ CFU/kg, and L. *plantarum* at 1 × 10^11^ CFU/kg) significantly increased VH compared with that in the control group (832 vs. 748 µm; *p* < 0.05). The LDP and HDP groups had a significantly higher CD but a significantly lower VH-to-CD ratio than did the other groups (*p* < 0.05). Incharoen et al. (2019) indicated that administering heat-killed L. *plantarum*l-137 at a concentration of 10 mg/kg basal diet increased CD (157.2 µm) but reduced the VH-to-CD ratio (5.5) on day 42 compared with corresponding values in control group (144.5 µm and 6.2, respectively; *p* = 0.503 and *p* = 0.523, respectively). Song et al. (2014) stated that a higher VH, a lower CD, and a higher VH-to-CD ratio are associated with higher intestinal integrity; an improvement in intestinal integrity increases the surface area available for nutrient absorption and mitigates enterocyte damage. In the present study, administering heat-killed probiotics significantly increased VH (HDP vs. CON: 13.43 %) and CD (HDP vs. CON: 26.21 %; LDP vs. CON: 17.18 %) and significantly reduced the VH-to-CD ratio (HDP vs. CON: 15.18 %; LDP vs. CON: 12.59 %). Taken together, these findings suggest that administering heat-killed probiotics at high concentrations increases not only nutrient absorption but also the intestinal tissue turnover rate. Despite the major changes observed in intestinal morphology in our study, no significant intergroup difference was noted in growth performance parameters. Al-Fataftah and Abdelqader (2014) reported that administering live BS at a concentration of 2.3 × 10^8^ CFU/g under thermoneutral conditions increased VH (14.84 %) and reduced CD (3.61 %) in broilers, without significantly affecting growth performance. In contrast, under heat stress, administering the same live BS at the same concentration significantly increased VH (24.76 %) and CD (7.73 %), which in turn improved BW, ADWG, and FCR. These findings suggest that the ratio of changes in intestinal morphological structure observed in this study may not have a significant impact on growth performance. Taking together, ratio difference in intestinal morphology could plays a crucial role to influence the growth performance in broilers.

In this study, hepatic GPx levels were significantly higher in the HDP group than in the other groups (*p* < 0.05), indicating an enhanced antioxidant capacity. This finding is consistent with those of Bai et al. (2017) and Xu et al. (2021), who reported that administering live BS significantly increased the production of antioxidant enzymes, such as GPx, SOD, catalase, and MDA, in broilers (*p* < 0.05). SOD, catalase, and GPx are type II antioxidant metabolic enzymes that eliminate excess reactive oxygen species by converting hydroxyl free radicals (^•^OH) and superoxide anions (O_2_^−^) into stable compounds such as water and oxygen (Fuller, 1989; Ko et al., 2004). Studies have indicated that natural additives such as probiotics induce the production of type II metabolic enzymes and regulate the function of these enzymes by activating the nuclear factor erythroid 2-related factor (Nrf2) pathway (Bai et al., 2017; Kode et al., 2008). Zhao et al. (2018) reported that live L. *plantarum* can activate the Nrf2 pathway, thereby regulating type II metabolic enzymes, such as catalase and SOD, in rats. In summary, probiotics promote the production of antioxidant enzymes, which scavenge reactive oxygen species and mitigate oxidative stress in broilers. In this study, no significant intergroup differences were observed in the serum or hepatic level of SOD or catalase. Moreover, no significant differences was noted in the serum level of GPx. TBARS levels were used as a major indicator of lipid peroxidation in meat (Yang et al., 2008). In the present study, TBARS levels in breast muscle were significantly lower in broilers fed a diet supplemented with heat-killed probiotics (LDP and HDP groups) than in the control group (CON group; *p* < 0.05; Table 4). Evidence suggests that probiotics prevent lipid peroxidation in muscle tissue by inducing the production of antioxidant enzymes, thereby improving the quality of meat (Kim et al., 2016; Sahu et al., 2008). Any increase in the hepatic level of the antioxidant enzyme GPx may mitigate oxidative stress in muscle tissue and reduce the levels of TBARS.

Meat quality in broilers is determined by various factors, such as physiochemical characteristics, meat appearance, and textural qualities. Unlike other treatment methods, probiotic-based diets do not alter the pH of breast meat. The standard pH value for broiler breast meat ranges from 5.9 to 6.1 (Beauclercq et al., 2022). In this study, the pH of breast meat in the CON group was 6.17 ± 0.10, suggesting dark, firm, and dry meat. However, the pH values in the probiotic-fed groups were between 6.07 and 6.10, which were within the normal range. Notably, pH is regarded as a key indicator of the physical characteristics of meat, particularly because it affects meat color, WHC, DL, CL, and texture (Devatkal et al., 2019). However, we noted no significant intergroup difference in DL, CL, or WHC. Meat color is a key factor that influences consumer preferences. In this study, the HDP group had a significantly higher b* value than did the LDP group (*p* < 0.05), indicating that administering high concentrations of heat-killed probiotics improved the appearance and reduced the yellowness of meat However, the probiotics treatments did not influence the meat lightness and redness compared with CON group in this study. Consistent with our findings, Zhang et al. (2012) and Sampath et al. (2021) demonstrated that adding live BS at a concentration of 1 × 10^8^ CFU/kg or live L. *plantarum* at a concentration of 0.10 % to the diet of broilers exerted no significant effect on pH, L*, a*, or WHC. In addition, similar results show the *B. subtilis* supplementation did not affect pH, meat color (L*, a*, and b*), DL, and WHC in broilers under *salmonella* challenge (Al-Owaimer et al., 2014).

In this study, shear force values were significantly lowered in the LDP and HDP groups than in the CON group (*p* < 0.05). Shear force is a key textural indicator of meat quality, related to the growth of skeletal muscle (Rødbotten et al., 2000). A lower shear force value is associated with greater meat tenderness (Liu et al., 2023). Probiotic-fed broilers typically have significantly high shear force values and high juiciness (*p* < 0.05; Yibar & Uzabaci, 2024; Zhou et al., 2010). Shear force is influenced by pH, which causes protein denaturation, thereby reducing shear force (Jin et al., 2021). Therefore, the low pH values observed in our probiotic-fed groups may be attributable to low shear force values. Meat quality indicators, such as hardness, springiness, and cohesiveness, were not alter among groups in this study. On the other hand, Our LDP and HDP groups exhibited significantly higher gumminess than did the CON group and significantly higher chewiness than did the CON and AB groups (*p* < 0.05). Meat tenderness, including gumminess and chewiness are primarily influenced by connective tissue, muscle fibers, and intramuscular fat in the meat (Mutia et al., 2022). Zhou et al. (2015) reported that administering a live probiotic mixture containing BS, *B. licheniformis*, and *B. subtilis* var. *natto* at concentrations of 200 and 400 mg/kg significantly increased meat tenderness by increasing the diameter of muscle fibers, reducing the amount of connective tissue, and increasing the cross links between muscle fibers (*p* < 0.05). Kim et al. (2016) indicated that administering a live probiotic mixture containing *Enterococcus, Pediococcus, Bifidobacterium*, and *Lactobacillus* species at a concentration of 1.0 × 10^8^ CFU/kg significantly increased the myofibrillar fragmentation index (*p* < 0.05), which in turn increased the tenderness of meat. Taken together, these findings suggest that probiotics influence the tenderness of meat by affecting the formation of muscle fibers. Overall, the present study suggests that heat-killed probiotic mixture not only improved intestinal morphology and antioxidant capacity, but also enhanced meat quality in broilers.

The main limitation of this study is that it involved only three replicates per treatment. Nevertheless, all broilers were maintained in a controlled environment, and all statistical power analyses were conducted (G power > 0.9) on the basis of pilot studies and the literature to ensure the reliability of the production outcomes. Overall, this study addresses the foundation for further research to explore the form of probiotic mixture affecting the broiler physiology and lead to affect the meat quality. Further studies are required to determine how heat-killed probiotics improve the antioxidant capacity and quality of meat.

## 5 Conclusion

Administering heat-killed probiotic mixtures containing LP28 and BS each at a concentration of 1.0 × 10^9^ cells/kg improves intestinal morphology and meat quality in broilers. However, it does not affect their growth performance. Probiotics increase the antioxidant capacity of the liver, thereby reducing lipid peroxidation in muscle tissue. Heat-killed probiotics improve the quality of meat by reducing shear force and increasing gumminess and chewiness. These findings suggest that heat-killed probiotics can be utilized not only as a stable form of feed during manufacturing procedures but also as an additive that improves intestinal health and meat quality in broilers.

## Funding statement

This study was financially funded by SYNBIO TECH INC., Kaohsiung, Taiwan.

## Ethical statement

The experiment was conducted on the research farm at National Pingtung University of Science and Technology (NPUST), Taiwan. All the animal experimental protocols were reviewed and approved by Institutional Animal Care and Use Committee (NPUST-111–083) of the university for the care and use of animals.

## CRediT authorship contribution statement

**Bishnu Prasad Bhattarai:** Writing – original draft, Visualization, Validation, Software, Methodology, Formal analysis, Data curation, Conceptualization. **Fu-Yuan Cheng:** Writing – review & editing, Supervision, Methodology, Investigation, Funding acquisition, Conceptualization. **Yu-Cheng Xu:** Methodology, Data curation. **Chi Yu:** Resources. **Ting-Yu Lee:** Resources, Methodology, Investigation, Funding acquisition, Formal analysis, Conceptualization. **Hsiao-Tung Chang:** Methodology, Investigation. **Hsiao-Ching Lin:** Resources, Methodology. **Hsiu-Ming Weng:** Methodology, Investigation. **Hsin-Hsuan Huang:** Resources, Methodology. **Jin-Seng Lin:** Validation, Investigation. **Chao-Wei Huang:** Writing – review & editing, Visualization, Validation, Supervision, Project administration, Methodology, Investigation, Funding acquisition, Conceptualization.

## Declaration of competing interest

The authors declare the following financial interests/personal relationships which may be considered as potential competing interests:

Chao-Wei Huang reports financial support was provided by SYNBIO TECH INC. If there are other authors, they declare that they have no known competing financial interests or personal relationships that could have appeared to influence the work reported in this paper.
